# Age-Related Effects of Exogenous Melatonin on Anxiety-like Behavior in C57/B6J Mice

**DOI:** 10.3390/biomedicines11061705

**Published:** 2023-06-13

**Authors:** Sofia Nasini, Sara Tidei, Atea Shkodra, Danilo De Gregorio, Marco Cambiaghi, Stefano Comai

**Affiliations:** 1 Department of Pharmaceutical and Pharmacological Sciences, University of Padova, 35131 Padova, Italy; sofia.nasini@phd.unipd.it (S.N.); saratidei@gmail.com (S.T.); 2 IRCCS San Raffaele Scientific Institute, 20132 Milan, Italy; shkodra.atea@hsr.it (A.S.); degregorio.danilo@hsr.it (D.D.G.); 3 School of Medicine, Vita Salute San Raffaele University, 20132 Milan, Italy; 4 Department of Neurosciences, Biomedicine and Movement Sciences, University of Verona, 37134 Verona, Italy; marco.cambiaghi@univr.it; 5 Department of Biomedical Sciences, University of Padova, 35131 Padova, Italy; 6 Department of Psychiatry, McGill University, Montreal, QC H3A 1A1, Canada

**Keywords:** melatonin, anxiety, mice, adolescents, adults, local field potentials, hippocampus, medial prefrontal cortex, sociability, aging

## Abstract

The synthesis of melatonin (MLT) physiologically decreases during aging. Treatment with MLT has shown anxiolytic, hypnotic, and analgesic effects, but little is known about possible age-dependent differences in its efficacy. Therefore, we studied the effects of MLT (20 mg/kg, intraperitoneal) on anxiety-like behavior (open field (OFT), elevated plus maze (EPMT), three-chamber sociability, and marble-burying (MBT) tests), and the medial prefrontal cortex (mPFC)-dorsal hippocampus (dHippo) circuit in adolescent (35–40 days old) and adult (three-five months old) C57BL/6 male mice. MLT did not show any effect in adolescents in the OFT and EPMT. In adults, compared to vehicles, it decreased locomotor activity and time spent in the center of the arena in the OFT and time spent in the open arms in the EPMT. In the MBT, no MLT effects were observed in both age groups. In the three-chamber sociability test, MLT decreased sociability and social novelty in adults, while it increased sociability in adolescents. Using local field potential recordings, we found higher mPFC-dHippo synchronization in the delta and low-theta frequency ranges in adults but not in adolescents after MLT treatment. Here, we show age-dependent differences in the effects of MLT in anxiety paradigms and in the modulation of the mPFC-dHippo circuit, indicating that when investigating the pharmacology of the MLT system, age can significantly impact the study outcomes.

## 1. Introduction

Anxiety disorders, including, among others, generalized anxiety disorder, social anxiety disorder, different phobias, obsessive-compulsive disorder, and panic disorder, are one of the major health problems of modern societies [1,2,3]. According to the World Health Organization, in 2019, more than 300 million people, of which 58 million were children and adolescents, were suffering from an anxiety disorder, and their prevalence increased by 26% in 2020 due to the COVID-19 pandemic [1]. The neurobiology of anxiety is still largely unknown [4,5], and the currently available medications, mostly benzodiazepines and antidepressants, induce several side effects and have many contraindications, especially when used in children, adolescents, and elderlies.

Melatonin (MLT) is a neuromodulator widely used in the world as an over-the-counter compound in both adolescents and adults for its anxiolytic and sedative/hypnotic properties despite the lack of clear evidence of activity [6,7]. MLT has shown anxiolytic-like effects in rodents in different behavioral paradigms of anxiety [8,9,10,11,12,13], as well as anxiolytic effects in humans, especially for reducing preoperative and postoperative anxiety in both children and adults [12,14,15]. The MLT system undergoes physiological changes from infants to adolescents, adults, and elderly. In particular, the synthesis of MLT in the pineal gland and, consequently, the peak of circulating levels occurring in the middle of the night is high between 5–10 years of age and then progressively declines with aging [16]. Although, as mentioned above, MLT is largely used in the population of all ages, and the MLT system undergoes changes according to aging, few preclinical and clinical studies have investigated possible age-dependent effects of MLT. MLT acts mainly by activating its two G-protein-coupled receptors named MT_1_ and MT_2_ [17], which display complementary or opposite effects in both the central nervous system and the periphery [18,19,20]. Concerning anxiety, preclinical data seem to indicate that the MLT receptor subtype most implicated is MT_2_ [10,21,22,23]. Indeed, it has been found that (1) the selective MT_2_ receptor’s partial agonist UCM765 induces anxiolytic-like effects in rats in different preclinical paradigms of anxiety similar to those of MLT, which are blocked by the selective MT_2_ antagonist 4P-PDOT [10]; (2) MT_2_ receptors in knockout mice display altered levels of behaviors related to the anxiety spectrum [21,22,24]; and (3) activation of MT_2_ receptors in the striatum produces anxiolytic-like effects in animal models of Parkinson’s disease, which is characterized by high comorbidity with anxiety disorders (more than 50% of the affected individuals) [23]. It is important to mention that while changes in circulating levels of MLT according to aging have been shown, there is no information on whether the expression of MT_1_ and MT_2_ receptors in the different regions of the brain may also change during development and aging. If this occurs, it is plausible that the pharmacological effects induced by MLT could also vary depending on aging. For this reason, in this work, we investigated whether treatment with MLT induced different effects in adolescent (35–40 day-old) and adult (3–4-month-old) male mice when tested in behavioral paradigms covering the spectrum of anxiety disorders and in the oscillatory synchrony between the medial prefrontal cortex (mPFC) and the dorsal hippocampus (dHippo), two regions of the brain involved in anxiety and widely expressing both MT_1_ and MT_2_ receptors [25,26]. We decided to test MLT at the dose of 20 mg/kg since we previously found—in male adult rats—that it had anxiolytic-like activity in the elevated plus maze test (EPMT) and novelty-suppressed feeding test (NFST) without inducing any effect in the open field test (OFT) [10].

## 2. Materials and Methods

### 2.1. Experimental Design

Mice were habituated to the testing room by transferring them to the testing room 30 min prior to the beginning of the trials. All behavioral and in vivo electrophysiology tests were performed between 9:00 am and 4:00 pm. After testing, each mouse was removed from the apparatus and returned to its home cage, and all interior surfaces were thoroughly cleaned with 70% ethanol and then wiped dry to remove any trace of conspecific odor. Three groups of mice per treatment (vehicle or MLT) and age (adolescents or adults) were used. One group was tested in the open field test and then one week later in the elevated plus maze test, whereas the second group was tested in the three-chamber sociability test and then one week later in the marble burying test. We left one week between the two tests in each group to minimize the possible effects of one test over the other and to have the washout from MLT. The animals were randomized to each experimental session for their treatment (vehicle or MLT). The behavior was videotaped using an LCD camera connected to control and recording equipment. Automated tracking of the mice was achieved using ANY-maze software (Stoelting Europe, Dublin, Ireland). The third group was used for in vivo local field potential (LFP) recordings after implanting the electrodes into the two brain regions of interest, the medial prefrontal cortex (mPFC) and the dorsal hippocampus (dHippo). At the time of the behavioral and electrophysiology analyses, the experimenter was blind to the treatment received by each individual mouse.

### 2.2. Animals

Male C57BL/6J mice used for these experiments were reared in breeding colonies of the Department of Pharmaceutical and Pharmacological Sciences (University of Padua). The animals were kept in a temperature-controlled room (22 °C) on a 12:12 h light–dark cycle (light on at 7:00 AM) and fed a standard pellet diet and tap water ad libitum. Mature adult mice ranged in age from 3 to 5 months (n = 10–15), and adolescent mice between 35 and 40 days of age (n = 10–15) were used for the experimental procedures. All experimental protocols were performed after authorization from the Animal Care and Use Ethics Committee of the University of Padova and the Italian Ministry of Health and were in compliance with national and European guidelines for the handling and use of experimental animals.

### 2.3. Treatment

MLT (CAS Number: 73-31-4, Cayman Chemical Co., Ann Arbor, MI, USA) was used at the dose of 20 mg/kg based on previous research [10,27], and it was dissolved in a vehicle composed of 30% saline and 70% dimethyl sulfoxide (DMSO; Sigma–Aldrich, Steinheim, Germany). Each mouse received a single intraperitoneal (I.P.) injection (total volume 0.1 mL) of vehicle or MLT (20 mg/kg) 10 min before each behavioral or in vivo electrophysiology test.

### 2.4. Behavioral Testing

#### 2.4.1. Open-Field Test (OFT)

The OFT to measure exploratory activity and locomotion was performed according to standardized protocols in the laboratory [28]. Briefly, mice were individually placed in the corner of a grey-painted open field arena (40 × 40 × 15 cm) and left to explore freely for 20 min. The experiment took place under standard room lighting (350 lx); a white lamp (100 W) was suspended 2 m above the arena. Anxiety-like behavior was measured by the frequency and total duration of visits to the central zone (20 × 20 cm) of the arena. Other ethological measures analyzed included grooming, rearing, and locomotor activity (total distance traveled).

#### 2.4.2. Elevated Plus Maze Test (EPMT)

The EPM to assess anxiety-related behaviors relies on rodents’ proclivity toward dark, enclosed spaces (approach) and an unconditioned fear of heights and open spaces (avoidance) [29]. It is plus-shaped, with two open arms (25 × 5 × 0.5 cm) and two enclosed arms (25 × 5 × 16 cm) with a central platform (5 × 5 × 0.5 cm). The closed arms are enclosed by two high walls (16 cm), whereas the open arms have no side wall. The apparatus was elevated to a height of 50 cm from the floor and weakly illuminated (350 lx). The walls of the enclosed arms were painted medium grey. Animals were placed in the center of the plus-maze and allowed to explore freely on the apparatus for 5 min. The time spent and number of entries into the open arm, as well as the time spent in the closed arm of the plus-maze, were measured according to our previous report [30].

#### 2.4.3. Three-Chamber Sociability Test

Social anxiety was measured following our previous method in the three-chamber sociability test [28,31]. In this test, mice were first left free to explore the three-chamber apparatus for 10 min. Then, in each of the lateral chambers, an up-turned metal-grid pencil cup was placed: one remained empty as the novel object (O), while an age- and sex-matched WT stranger mouse (S1) was placed in the second up-turned cup. The stranger mouse was previously habituated to the cups for 3 × 10 min sessions. The testing mouse was left 10 min to explore the apparatus and to interact with either O or S1 (Sociability phase). Finally, to test for social preference, mice were presented for another 10 min with the choice of object (O), which now contained a second age- and sex-matched stranger mouse or the now familiar mouse (S1). Sociability and social novelty were determined manually by assessing the time spent by the tested mouse actively interacting with O or S1 in the sociability and social novelty phases, respectively.

#### 2.4.4. Marble Burying Test

Marble burying test is used to depict anxiety or obsessive–compulsive disorder (OCD) behavior. It is based on the observation that mice will bury either harmful or harmless objects in their bedding [32]. This behavior is a correlational model for the detection of anxiolytics rather than an isomorphic model of anxiety [33]. Each mouse was placed in a cage filled approximately 5 cm deep with wood chip bedding, lightly tamped to make a flat, even surface, and left there for 30 min for habituation. Twenty glass marbles were then placed in a regular pattern, evenly spaced. The number of marbles buried (for at least 2/3 of the area) with bedding was counted to measure the obsessive–compulsive behavior.

### 2.5. In Vivo LFP Recordings and Analysis

Extracellular field potentials were recorded in freely moving mice in a 20 × 30 × 30 cm box 10 min after vehicle or 20 mg/kg MLT I.P. injection to examine the oscillatory synchrony between the mPFC and the dHippo in the two conditions. Following a standard procedure in the laboratory [34], stainless steel insulated wires (∅ 135 μm) were stereotaxically implanted unilaterally (right side) in the mPFC and the dHippo according to the following coordinates, in mm from bregma: mPFC, +1.8 AP, 0.3 ML, −2.4 DV and hippocampus, −2.1 AP, 1.5 ML, −1.4 DV. A screw over contralateral parietal areas served as a common reference and ground. All implants were secured using dental cement (Ketacem). After surgery, mice were allowed to recover for 5–6 days before testing [35]. LFPs were recorded and initially digitalized at 1 kHz and stored on a hard drive for offline analysis. LFP epochs were visually examined, and artifact-free segments were computed by analyzing 3 segments of 2 s each during the recording sessions after both vehicle and MLT 20 mg/kg injections. The coherence between LFP channels was measured by magnitude squared coherence (MSC), using the function mscohere in Matlab signal toolbox, which is a coherence estimate of the input signals x and y by using Welch’s averaged, modified periodogram method. The MSC estimate is a function of frequency with values between 0 and 1 and indicates how well x corresponds to y at each frequency. The MSC estimate was calculated over the frequency range of 0.5–30 Hz for each mouse with a frequency resolution of 0.5 Hz. To test whether coherence values were significantly higher than expected by chance, we performed a permutation test in which coherence values were compared before inclusion in additional analyses with a shuffle procedure in which epochs were randomly shifted 5–10 s relative to each other. This process was repeated 1000 times to obtain the distribution of coherence expected by chance [34]. Differences in coherence were obtained by comparing coherence values (20 mg/kg MLT vs. vehicle), and statistics were performed on the normalized coherence within the frequency bands of interest: delta (1–4 Hz), low-theta (4–8 Hz), high-theta (8–12 Hz), beta (12–30 Hz) [34].

### 2.6. Statistical Analysis

Statistical analyses were conducted using GraphPad 8.0 (GraphPad Software, La Jolla, CA, USA) software. The normal distribution of data was verified with the Shapiro–Wilk test. Two-tailed unpaired Student’s *t*-test was performed for the Open Field test, stereotypic behaviors, EPM, MBT, and coherence bands. Two-way ANOVA for repeated measures followed by Bonferroni post-hoc analysis was used for the three-chamber test. *p* < 0.05 was considered statistically significant. Data were presented as mean ± SEM. Table 1 and Table 2 report statistical details for the different experiments in adolescent and adult mice, respectively.

## 3. Results

### 3.1. Adolescent Mice

#### 3.1.1. Evaluation of the Effects of MLT on Anxiety-like Behaviors: OFT, Stereotypic Behaviors and EPMT

**Figure 1:**
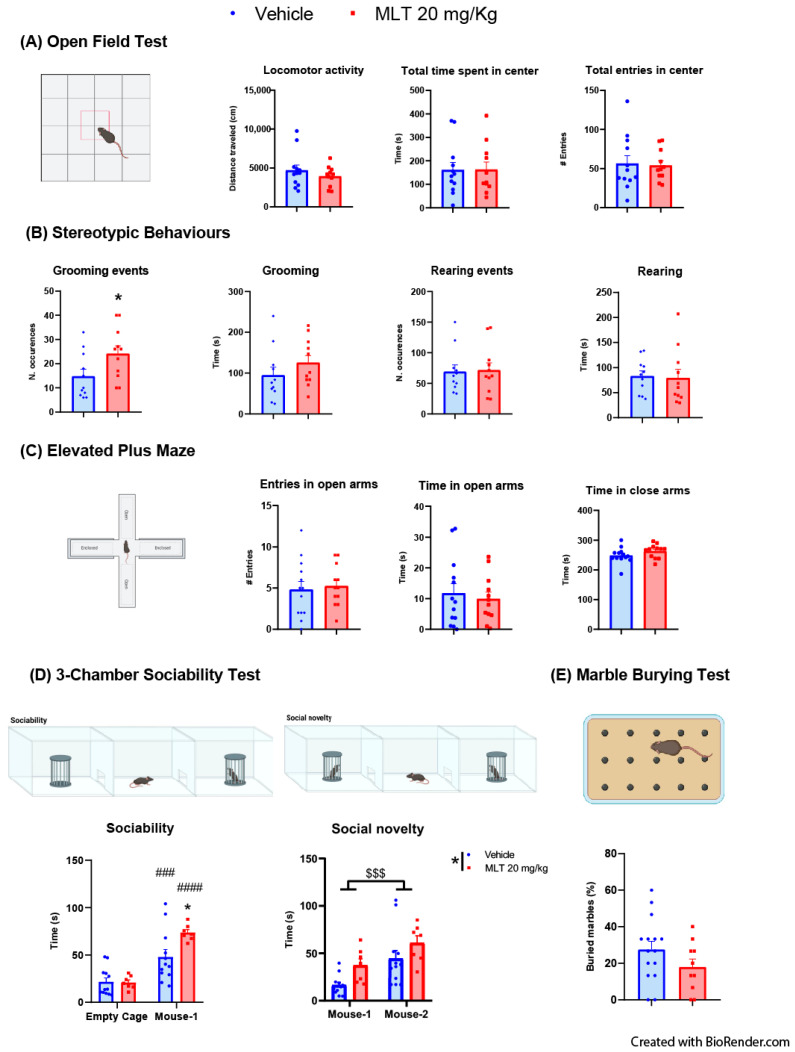
Behavioral testing in adolescent mice performed 10 min after the treatment with vehicle or 20 mg/kg melatonin (MLT). (**A**) Open Field test: the locomotor activity, the time spent in the central zone of the arena, and the number of entries in the center of the arena were evaluated during 20 min. (**B**) The number of occurrences and the duration of grooming and rearing (stereotypic behaviors) were measured during the 20 min Open Field test. Grooming events: statistical analyses were performed using the two-tailed unpaired Student’s *t*-test; * *p* < 0.05 vehicle vs. 20 mg/kg. (**C**) Elevated Plus Maze test: effect of vehicle or MLT (20 mg/kg) on the number of entries in the open arms, the time spent in open arms, and the time spent in closed arms. (**D**) Three-Chamber Sociability test: the interaction time of mice treated with vehicle or MLT (20 mg/kg) was measured during both the sociability (empty cage vs. cage with an unfamiliar conspecific mouse (mouse 1)) and social novelty (cage with the familiar conspecific mouse from the previous phase (mouse 1) vs. cage with a second unfamiliar conspecific mouse (mouse 2)). Statistical analyses were performed using the two-way ANOVA for repeated measures followed by Bonferroni’s multiple comparison test; * *p* < 0.05 vehicle vs. MLT 20 mg/kg, ### *p* < 0.001 vehicle (empty cage vs. mouse-1), #### *p* < 0.0001 MLT 20 mg/kg (empty cage vs. mouse-1), $$$ *p* < 0.001 social novelty (mouse-1 vs. mouse-2). (**E**) Marble Burying test: the number of marbles buried during the 30 min test were measured, and the percentage of buried marbles was calculated. All datasets are represented as mean ± SEM; individual mice are represented as dots (vehicle) or squares (MLT).

Evaluation of anxiety-like behavior was first conducted using the OFT, in which we also observed two stereotypic behaviors, grooming, and rearing. In adolescent mice (Figure 1A), the two-tailed unpaired Student’s *t*-test showed no differences between mice treated with vehicle and 20 mg/kg MLT in the locomotor activity, in the time spent in the center of the arena and in the total number of entries in the center. The number of grooming events was higher in 20 mg/kg MLT-treated adolescents; instead, there was no difference in the number of rearing events. The total duration of both grooming and rearing events did not vary between adolescent mice treated with vehicle or 20 mg/kg MLT (Figure 1B).

The EPMT allows evaluation of the possible anxiolytic-like activity of a psychoactive compound by determining the time spent in the open arms of the apparatus and the number of entries in the open arms that represent a place where mice feel exposed to danger. No effects of 20 mg/kg MLT were observed concerning the time spent in open and closed arms and the number of entries in the open arms (Figure 1C).

#### 3.1.2. Evaluation of the Effects of MLT on Sociability: Three-Chambers Sociability Test

In adolescents (Figure 1D), the two-way ANOVA for repeated measures analysis for the sociability stage resulted in a significant interaction between treatment and sociability and an effect of sociability, but no effect of treatment with both groups of animals spending more time interacting with the novel animal than the empty cage. Furthermore, MLT-treated mice interacted for a longer time with the novel animal than vehicle-treated mice. In the second stage of the test, there was no interaction between factors, but there was an effect of the social novelty, as both MLT-treated mice and vehicle-treated mice interacted more with the mouse-2 than mouse-1; moreover, there was an effect of treatment with animals treated with MLT interacting longer with both mouse 1 and mouse 2 than mice receiving vehicle.

#### 3.1.3. Evaluation of the Effects of MLT on Obsessive–Compulsive Disorder (OCD) Behavior: Marble Burying Test

Obsessive–compulsive behavior, represented by repetitive actions, can be evaluated through the Marble Burying test by calculating the percentage of marbles buried. No statistically significant difference in the percent of marbles buried emerged from the two-tailed unpaired Student’s *t*-test between vehicle-treated and 20 mg/kg MLT-treated mice (Figure 1E).

#### 3.1.4. In Vivo Electrophysiology

**Figure 2:**
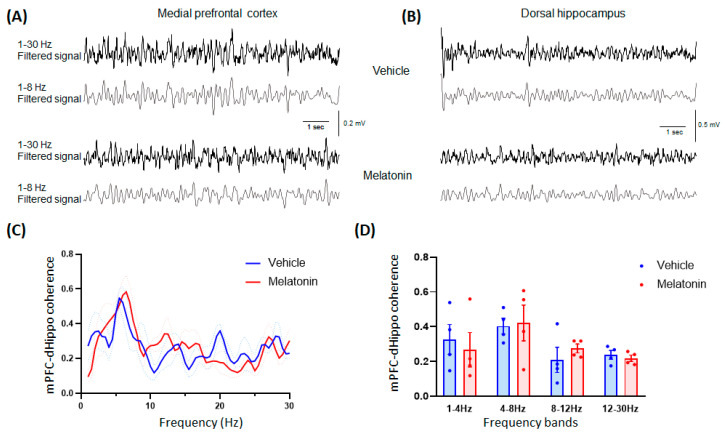
Medial prefrontal cortex (mPFC)-dorsal hippocampus (dHippo) synchrony in adolescent mice. Example of LFP traces (1–30 Hz filtered signal in black; 1–8 Hz filtered signal in grey) recorded in mPFC (**A**) and in dHippo (**B**) of an adolescent mouse 10 min after the treatment with vehicle (top) or 20 mg/kg melatonin (bottom). No effect of the treatment with MLT was observed in (**C**) the mPFC-dHippo coherence spectrum and in (**D**) all the analyzed frequency bands. Data are represented as mean ± SEM (n = 4).

A coherence analysis based on LFP recordings was used to measure the functional connectivity among different brain areas. We measured the effects of vehicle and MLT on the mPFC-dHippo synchrony in the adolescent mice (Figure 2A,B) and observed no overall changes in the spectrum due to MLT treatment but higher coherence values in the low-theta range in both vehicle and MLT treated mice (Figure 2C). Moreover, no difference between treatment with vehicle and MLT was found in the coherence levels for all the examined frequency bands (Figure 2D).

### 3.2. Adult Mice

#### 3.2.1. Evaluation of the Effects of MLT on Anxiety-like Behaviors: OFT, Stereotypic Behaviors and Epmt

**Figure 3:**
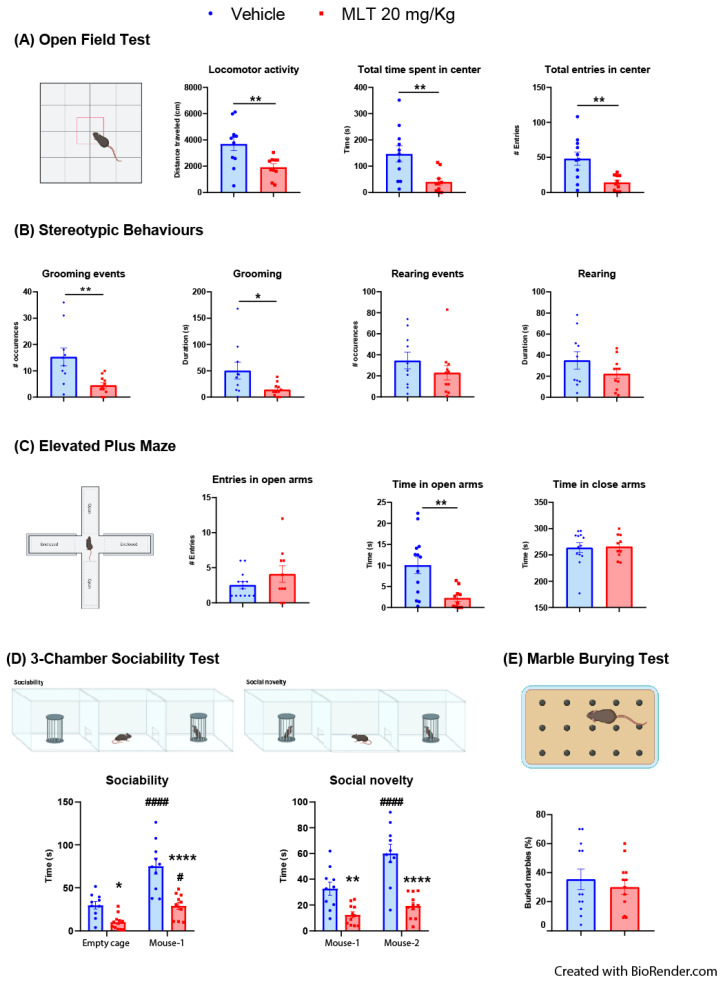
Behavioral testing in adult mice performed 10 min after the treatment with vehicle or 20 mg/kg melatonin (MLT). (**A**) Open Field test: the locomotor activity, the time spent in the central zone of the arena, and the number of entries in the center, were evaluated over 20 min. Statistical analyses were performed using the two-tailed unpaired Student’s *t*-test; ** *p* < 0.01 vehicle vs. 20 mg/kg MLT. (**B**) The number of occurrences and the duration of grooming and rearing (stereotypic behaviors) were measured during the 20 min of the Open Field test. Statistical analyses were performed using the two-tailed unpaired Student’s *t*-test; * *p* < 0.05, ** *p* < 0.01 vehicle vs. 20 mg/kg MLT. (**C**) Elevated Plus Maze test: effect of vehicle or MLT (20 mg/kg) on the number of entries in the open arms, the time spent in open arms, and the time spent in closed arms. Statistical analyses were performed using the two-tailed unpaired Student’s *t*-test; ** *p* < 0.01 vehicle vs. 20 mg/kg MLT. (**D**) Three-Chamber Sociability test: the interaction time of the mice treated with vehicle or MLT (20 mg/kg) was measured during both the sociability (empty cage vs. cage with an unfamiliar conspecific mouse (mouse 1)) and social novelty (cage with the familiar conspecific mouse from the previous phase (mouse 1) vs. cage with a second unfamiliar conspecific mouse (mouse 2)). Statistical analyses were performed using the two-way ANOVA for repeated measures followed by Bonferroni’s multiple comparison test; * *p* < 0.05, ** *p* < 0.01, **** *p* < 0.0001 vehicle vs. 20 mg/kg MLT; # *p* < 0.05, #### *p* < 0.0001 empty cage vs. mouse 1, or mouse 1 vs. mouse 2. (**E**) Marble Burying test: the number of marbles buried during the 30 min of the test were measured, and the percentage of buried marbles was calculated. All datasets are represented as ± SEM, individual mice are represented as dots (vehicle) or squares (MLT).

In the OFT (Figure 3A), adult mice treated with 20 mg/kg MLT displayed lower locomotor activity, a lower time spent in the central zone of the arena, and a lower number of entries in the center compared to adult mice treated with vehicle. These results indicate that MLT at the dose of 20 mg/kg in our experimental conditions induced a sedative-like state in adult mice. Adult animals (Figure 3B) treated with 20 mg/kg MLT decreased the number (and the duration of grooming events) in keeping with a sedative-like activity of MLT at the tested dose. On the other hand, the number and duration of rearing events were not significantly affected by 20 mg/kg MLT.

In the EPMT, adult mice (Figure 3C) treated with 20 mg/kg MLT spent significantly less time in the open arms with respect to vehicle-treated mice. The number of entries in the open arms and the time spent in closed arms were not significant comparing adult mice treated with vehicle and MLT (20 mg/kg).

#### 3.2.2. Evaluation of the Effects of MLT on Sociability: Three-Chambers Sociability Test

Two-way ANOVA for repeated measures analysis of sociability in adult mice (Figure 3D) showed an interaction between treatment and sociability and an effect of treatment and sociability. The 20 mg/kg MLT significantly reduced the time of interaction with the empty cage as well as the time of interaction with the familiar mouse compared to vehicle-treated mice. However, we found that both mice treated with vehicle and 20 mg/kg MLT interacted longer with mouse 1 than with the empty cage. In the social novelty stage, the two-way ANOVA for repeated measures analysis resulted in an interaction between treatment x social novelty and an effect of treatment and social novelty. In particular, we observed a reduction in the time of interaction of MLT-treated mice with familiar and unfamiliar mice compared to vehicle-treated mice. Finally, unlike mice treated with 20 mg/kg MLT, we found that mice treated with vehicle interacted significantly longer with mouse 2 than mouse 1.

#### 3.2.3. Evaluation of the Effects of MLT on Obsessive–Compulsive Disorder (OCD) Behavior: Marble Burying Test

We did not find any effect of 20 mg/kg MLT on the percentage of marble buried by the mice during the 30 min test (Figure 3E).

#### 3.2.4. In Vivo Electrophysiology

**Figure 4:**
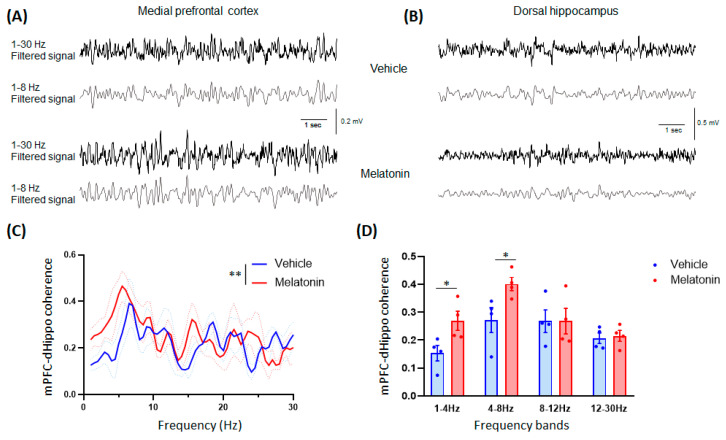
Medial prefrontal cortex (mPFC)–dorsal hippocampus (dHippo) synchrony in adult mice. Example of LFP traces (1–30 Hz filtered signal in black; 1–8 Hz filtered signal in grey) recorded in mPFC (**A**) and in dHippo (**B**) of an adult mouse 10 min after the treatment with vehicle (top) or 20 mg/kg melatonin (bottom). (**C**) A significant effect due to MLT treatment was observed in mPFC-dHippo coherence spectrum (two-way ANOVA, effect of treatment: F(1,354) = 9.304, *p* = 0.0025; ** *p* < 0.01 vehicle vs. 20 mg/kg MLT). (**D**) Melatonin induced a significant increase in coherence in the delta and low-theta frequency bands. Data are represented as mean ± SEM (n = 4). Statistical analyses were performed using the two-tailed unpaired Student’s *t*-test; * *p* < 0.05 vehicle vs. 20 mg/kg MLT.

In adult mice, the effects of MLT on mPFC-dHippo synchronization led to an increased coherence at low frequencies below 10 Hz (Figure 4A–C). Indeed, compared with vehicle, the MLT treatment resulted in a more synchronized activity in the mPFC and dHippo at delta and lower theta frequencies (Figure 4D).

## 4. Discussion

In this study, we examined whether treatment with MLT could affect different aspects of anxiety-like phenotype according to age. Using adolescent (35–40 days of age) and adult (3–4 months of age) mice, we found that MLT, at a dose of 20 mg/kg, had different effects on behavioral paradigms of anxiety-like behaviors according to age. We found that in adolescent mice, 20 mg/kg MLT had no effect in the OFT, EPMT, and Marble Burying test and increased social behavior in the Three-Chamber Sociability test. On the contrary, in adult mice, 20 mg/kg MLT reduced the distance traveled in the OFT, an indication of a sedative effect induced by the drug at this dose. This sedative effect was also reflected in the reduced exploration of the center of the open field arena in the OFT, of the open arms of the EPMT, and in the overall social encounters in the Three-Chamber Sociability test. Similar to the adolescents, 20 mg/kg MLT in adults did not alter the number of marbles buried in the Marble Burying test. These distinct behavioral age-dependent effects induced by MLT were also paralleled by differences in activation of the mPFC-dHippo brain regions. Using LFP recordings in freely moving animals, we found that 20 mg/kg MLT induced a significant increase in mPFC-dHippo coherence in the low-frequency bands (delta and low theta) in adults but not in adolescents.

Previous research, in keeping with our findings, has shown different effects induced by exogenous administration of MLT according to age. Sharman et al. [36] found that treatment with MLT in aged mice was able to reverse the changes in the expression levels of various genes associated with inflammation and immune function, including lipocalin 2 mRNA, to the levels observed in younger mice. The same research group [37] also analyzed the expression levels of these genes following an inflammatory insult by lipopolysaccharide and found that MLT treatment was able to induce a pattern of response in the gene expression in the brain of aged mice that mirrored that of younger mice.

As already mentioned, we chose the dose of 20 mg/kg according to our previous study in adult rats, in which it induced anxiolytic-like effects in the EPMT and novelty-suppressed feeding test without affecting locomotor behavior [10]. Unlike our expectations, we found that in adult C57BL6/J mice, 20 mg/kg MLT induced a sedative-like effect, as measured by reduced locomotor activity in the OFT. It is noteworthy that C57 mice are characterized by a low synthesis of MLT in the pineal gland caused by a natural point mutation in the gene encoding for the enzyme aralkylamine N-acetyltransferase (AANAT), which transforms serotonin into N-acetyl serotonin (NAS) that is then converted into MLT by the enzyme hydroxy-o-methyltransferase [38]. However, they found that serotonin can be acetylated by arylamine transferase (NAT), an alternative to the AANAT enzyme [39,40], leading to the production of NAS in C57BL/6 mice despite genetic defects in AANAT. Of interest, redundancy in serotonin acetylation was also observed on the skin of hamsters [41], rats [42], and humans [43], and NAS can be detected in human serum [44]. Thus, we can hypothesize that the low circulating levels of MLT, along with a blunting of the daily circadian variation in its levels in C57 mice compared to rats, make the MLT receptors more sensitive to an exogenous injection of MLT in C57 mice than in rats. This translates to the fact that an anxiolytic dose in adult rats instead induced sedation in adult C57 mice, a condition characterized by a slightly more profound depressive state of the central nervous system. In keeping with a higher dose of MLT, 40 mg/kg induced sleep when administered to adult rats [45], while a lower dose (10 mg/kg) did not reduce the total distance traveled in adult C57 mice in the OFT (data not reported). However, future studies should confirm these hypotheses by showing dose-response curves after MLT treatment in the different behavioral tests and by investigating the age-dependent effects of MLT in other strains of mice, the so-called melatonin-proficient mice, including C3H and CBA, or the relatively newly developed Aanat+/+; Hiomt+/+ on the C57BL/6J genetic background model [46,47].

Although it has not been fully clarified yet, MT_1_ and MT_2_ receptors have been reported to desensitize according to circulating levels of MLT [48,49,50]. Therefore, it can be hypothesized that the effects of exogenous MLT may depend on the intrinsic status of MLT receptors. In line with this hypothesis, we and others have demonstrated in preclinical and clinical studies that the response to an exogenous injection of MLT or a melatonergic compound can vary according to the time of the day [19,45,51,52,53]. It is well known that the levels of circulating MLT change dramatically during development and aging, but whether also the expression of MLT receptors and their intrinsic functioning vary during development and with aging is yet to be elucidated. The fact that a dose of MLT inducing sedative-like effects in adults did not alter the explorative behavior of the adolescent mice, but enhanced social encounters with peers, can therefore be viewed in the different intrinsic functioning of the endogenous MLT system (MLT production, expression, and functioning of its receptors) between adolescents and adults. Future studies are needed to support this hypothesis and should investigate whether changes in the expression and function of MLT receptors in different brain regions can occur during development and aging. At the same time, pharmacokinetic aspects should also be considered, given that the absorption, distribution, metabolism, and excretion of drugs are known to undergo major changes during aging [54]. In our experimental conditions, given that we injected MLT intraperitoneally, it is unlikely that differences in the absorption of MLT occurred. In contrast, we cannot exclude the possibility that differences in the distribution, metabolism, and excretion of MLT could be present comparing adolescent and adult mice. Future studies measuring the levels of MLT, for example, in the brain, specifically in the mPFC and dHippo, may clarify these aspects.

Among the different symptoms of social anxiety disorder, there is an intense fear of interacting with strangers [55]. We assessed social anxiety using the Three-Chamber Sociability test, which is also used to study autism spectrum disorders (ASD). A high comorbidity between social anxiety disorder and ASD has been reported [56,57], and MLT is a compound largely used in individuals with ASD, as it seems to ameliorate their sleep dysfunctions, where improvement leads to better functioning during the day [58]. Our data seem to support this evidence mainly in adolescents since we found an increase in social encounters in the Three-Chamber Sociability test after treatment with MLT. In keeping, a recent study using the valproic acid mouse model of ASD showed that MLT was rescuing social deficits in the three-chamber test of 6-week-old valproic acid-exposed offspring [59], and another one using the same ASD model but in rats showed an improvement in social deficits given by agomelatine, a non-selective MT_1_-MT_2_ receptor agonist and a 5HT_2C_ receptor antagonist [60]. However, these studies did not investigate which one of the two MLT receptors could be involved in the prosocial effects of MLT. A study by Thomson et al. [22] examining the phenotype of MT_2_ receptor knockout mice found that male mice had increased sociability compared to wild-type, highlighting a possible role for MT_2_ receptors in the modulation of social behavior.

Collectively, this preclinical evidence, further supported by our findings, indicates that MLT may not only have efficacy in ASD due to its modulation of the sleep-wake cycle and circadian system [58], but also a direct effect on regulating social behavior cannot be excluded.

Age-dependent differences in the behavioral effects induced by MLT were paralleled by differences in the activation of the mPFC-dHippo crosstalk, as highlighted by the analysis of LFP synchrony of freely moving animals. While in adolescent mice, no significant differences were observed after MLT treatment, in the coherence between the mPFC and the dHippo, in adult mice, we found an increased synchronization in the lower frequencies, corresponding to delta and low-theta bands. Although mPFC is known to be highly correlated with the ventral hippocampus in anxiety [61], we opted to record the correlation between the mPFC and the dHippo mainly due to (1) the high presence of MLT receptors in both the mPFC and the rostral rather than the ventral region of the hippocampus [25,26], (2) the fact that preliminary work has suggested a possible role for the dHippo in the anxiolytic effects of MLT [62], (3) acting on the process of memory reconsolidation in which the dHippo plays a major role [63] and appears to be a novel promising strategy for treating anxiety disorders [64]. Remarkably, recent studies have demonstrated that direct functional projections from the dorsal hippocampus to the prelimbic cortex are necessary for strengthening aversive memory through different molecular mechanisms [65,66], while increased theta coherence is observed during spatial memory tasks and after the application of dopamine in mPFC [67]. In a mouse model of schizophrenia, dHippo-mPFC theta coherence was impaired during working memory performance [68], similar to the altered functional connectivity between the frontal and temporal lobes observed in patients with schizophrenia. Different findings suggest that a coupling within the low frequencies is observed between the medial frontal cortex and distant brain regions to guide behavioral performances. In particular, the mPFC seems to play a key role in regulating social and emotional-like behavior and also responses to stress and fear, recruiting cortical and subcortical areas within the low-frequency range [69,70].

Therefore, an important future step will be to examine—in a more comprehensive way—the effects of MLT during cognitive/behavioral tasks in association with a functional study of brain-related regions. In particular, these studies should be performed considering the factor of age, as multiple evidence has shown that the neural basis underlying the neurobiology of anxiety and emotional processing can be different when comparing adults and adolescents [71,72,73,74]. Accordingly, as we found, the modulatory effects of MLT on anxiety circuits may expect to vary during development and aging.

## 5. Conclusions

Here, we show that exogenous MLT may affect anxiety-like behaviors in a different manner according to age. In particular, it seems that adult mice may be more sensitive than adolescent mice to the pharmacological effects of MLT, as also evidenced by a different functional modulation of the prefrontal-hippocampal circuit. Given the significantly increased prevalence in the use of MLT supplements during the last two decades [75], and the lack of clinical studies in adolescents and analyzing the outcomes according to the age of the participants, our findings highlight the need to take into account the age factor when evaluating the therapeutics and the toxicity of MLT or MLT receptor ligands in both preclinical and clinical studies.

## Figures and Tables

**Table 1:** Statistical details for the behavioral and in vivo electrophysiology experimental comparisons between vehicle- and 20 mg/kg MLT-treated adolescent mice.

Figure	Panel		Test	Group-Size	Statistic	*p* Value	Pair-Wise Comparison	Statistic 2		
1	A	Open field test	Student’s *t*-test	Vehicle = 11 mice	locomotor activity t = 0.9675; df = 21	*p* = 0.3443	N/A	N/A		
					time spent in centre t = 0.03147; df = 21	*p* = 0.9752				
				MLT 20 mg/kg = 10 mice	entries in centre t = 0.193; df = 21	*p* = 0.8488				
	B	Stereotypic behaviours	Student’s *t*-test	Vehicle = 11 mice	grooming events t = 2.18; df = 20	*p* = 0.0414	N/A	N/A		
					time of grooming t = 1.167; df = 20	*p* = 0.257				
				MLT 20 mg/kg = 11 mice	rearing events t = 0.1577; df = 20	*p* = 0.8763				
					time of rearing t = 0.1773; df = 20	*p* = 0.8610				
	C	Elevated Plus Maze	Student’s *t*-test	Vehicle = 13 mice	entries in open arms t = 0.3292; df = 23	*p* = 0.745	N/A	N/A		
				MLT 20 mg/kg = 12 mice	time in open arms t = 0.4702; df = 23	*p* = 0.6427				
					time in close arms t = 1.469; df = 23	*p* = 0.1553				
	D	3-chamber sociability test	Two-way ANOVA	Vehicle = 12 mice	Sociability:			**Test Details**	**t**	***p* Value**
					interaction F (1,17) = 8.171	*p* = 0.0109	Bonferroni post hoc comparison	empty cage vehicle vs. empty cage MLT 20 mg/kg	0.0695	*p* > 0.9999
					treatment F(1,17) = 2.886	*p* = 0.1076		mouse-1 vehicle vs. mouse-1 MLT 20 mg/kg	2.953	*p* = 0.0113
					sociability F (1,17) = 73.49	*p* < 0.0001		vehicle empty cage vs. vehicle mouse-1	4.707	*p* = 0.0004
								MLT 20 mg/kg empty cage vs. MLT 20 m/kg mouse-1	7.192	*p* < 0.0001
				MLT 20 mg/kg = 7 mice	Social novelty:		N/A	N/A		
					interaction F (1,17) = 0.1712	*p* = 0.6842				
					treatment F (1,17) = 4.570	*p* = 0.0474				
					social novelty F (1,17) = 22.93	*p* < 0.0001				
	E	Marble burying test	Student’s *t*-test	Vehicle = 15 mice	t = 1.454; df = 23	*p* = 0.1594	N/A	N/A		
				MLT 20 mg/kg = 10 mice						
2	C	mPFC-dHippo coherence	Two-way ANOVA	Vehicle = 4 mice	interaction F (58,354) = 0.8933	*p* = 0.6937	N/A	N/A		
					frequency F (58,354) = 3.410	*p* < 0.0001				
				MLT 20 mg/kg = 4 mice	treatment F (58,354) = 0.1881	*p* = 0.6648				
	D	mPFC-dHippo coherence	Student’s *t*-test	Vehicle = 4 mice	1–4 Hz t = 0.4544; df = 6	*p* = 0.6655	N/A	N/A		
					4–8 Hz t = 0.1669; df = 6	*p* = 0.8729				
				MLT 20 mg/kg = 4 mice	8–12 Hz t = 0.8479; df = 6	*p* = 0.4290				
					12–30 Hz t = 0.6128; df = 6	*p* = 0.5625				

**Table 2:** Statistical details for the behavioral and in vivo electrophysiology experimental comparisons between vehicle- and 20 mg/kg MLT-treated adult mice.

Figure	Panel		Test	Group-Size	Statistic	*p* Value	Pair-Wise Comparison	Statistic 2		
3	A	Open field test	Student’s *t*-test	Vehicle = 11 mice	locomotor activity t = 3.011; df = 19	*p* = 0.0072	N/A	N/A		
					time spent in centre t = 3.096; df = 19	*p* = 0.006				
				MLT 20 mg/kg = 10 mice	entries in centre t = 3.319; df = 19	*p* = 0.0036				
	B	Stereotypic behaviours	Student’s *t*-test	Vehicle = 10 mice	grooming events t = 3.138; df = 19	*p* = 0.0054	N/A	N/A		
					time of grooming t = 2.336; df = 19	*p* = 0.0306				
				MLT 20 mg/kg = 11 mice	rearing events t = 1.102; df = 19	*p* = 0.2843				
					time of rearing t = 1.377; df = 19	*p* = 0.1844				
	C	Elevated Plus Maze	Student’s *t*-test	Vehicle = 13 mice;	entries in open arms t = 1.32; df = 21	*p* = 0.2011	N/A	N/A		
				MLT 20 mg/kg = 10 mice	time in open arms t = 3.260; df = 21	*p* = 0.0037				
					time in close arms t = 0.177; df = 21	*p* = 0.8612				
	D	3-chamber sociability test	Two-way ANOVA	Vehicle = 11 mice	Sociability:			**Test Details**	**t**	***p* Value**
					interaction F(1,19) = 9.176	*p* = 0.0069	Bonferroni post hoc comparison	vehicle empty cage vs. vehicle mouse-1	7.172	*p* < 0.0001
					treatment F(1,19) = 26.29	*p* < 0.0001		MLT 20 mg/kg empty cage vs. MLT 20 mg/kg mouse-1	3.132	*p* = 0.0110
					sociability F(1,19) = 54.06	*p* < 0.0001		empty cage vehicle vs. empty cage MLT 20 mg/kg	2.53	*p* = 0.0314
								mouse-1 vehicle vs mouse-1 MLT 20 mg/kg	5.943	*p* < 0.0001
				MLT 20 mg/kg = 11 mice	Social novelty:			**Test Details**	**t**	***p* Value**
					interaction F(1,19) = 9.559	*p* = 0.006	Bonferroni post hoc comparison	vehicle mouse-1 vs. vehicle mouse-2	5.729	*p* < 0.0001
					treatment F(1,19) = 26.46	*p* < 0.0001		MLT 20 mg/kg mouse-1 vs. MLT 20 mg/kg mouse-2	1.529	*p* = 0.2857
					social novelty F(1,19) = 27.06	*p* < 0.0001		mouse-1 vehicle vs. mouse-1 MLT 20 mg/kg	3.11	*p* = 0.0071
								mouse-2 vehicle vs. mouse-2 MLT 20 mg/kg	6.246	*p* < 0.0001
	E	Marble burying test	Student’s *t*-test	Vehicle = 12 mice	t = 0.6125; df = 22	*p* = 0.5465	N/A		N/A	
				MLT 20 mg/kg = 12 mice						
4	C	mPFC-dHippo coherence	Two-way ANOVA	Vehicle = 4 mice	interaction F (1,354)	*p* = 0.3028	N/A		N/A	
					frequency F (1,354)	*p* = 0.0008				
				MLT 20 mg/kg = 4 mice	treatment F (1,354)	*p* = 0.0025				
	D	mPFC-dHippo coherence	Student’s *t*-test	Vehicle = 4 mice	1–4 Hz t = 2.613; df = 6	*p* = 0.04	N/A		N/A	
					4–8 Hz t = 2.553; df = 6	*p* = 0.0433				
				MLT 20 mg/kg = 4 mice	8–12 Hz t = 0.001256; df = 6	*p* = 0.9990				
					12–30 Hz t = 0.3003; df = 6	*p* = 0.7741				

## Data Availability

The data presented in this study are available upon reasonable request from the corresponding author.
